# A New Statistical Approach for the Evaluation of Gap-prepulse Inhibition of the Acoustic Startle Reflex (GPIAS) for Tinnitus Assessment

**DOI:** 10.3389/fnbeh.2017.00198

**Published:** 2017-10-18

**Authors:** Achim Schilling, Patrick Krauss, Richard Gerum, Claus Metzner, Konstantin Tziridis, Holger Schulze

**Affiliations:** ^1^Experimental Otolaryngology, ENT Hospital, Head and Neck Surgery, Friedrich-Alexander University Erlangen-Nürnberg, Erlangen, Germany; ^2^Biophysics Group, Department of Physics, Center for Medical Physics and Technology, Friedrich-Alexander University Erlangen-Nürnberg, Erlangen, Germany

**Keywords:** hearing loss, Bonferroni correction, tinnitus screening, Turner paradigm, effect size

## Abstract

**Background:** An increasingly used behavioral paradigm for the objective assessment of a possible tinnitus percept in animal models has been proposed by Turner and coworkers in 2006. It is based on gap-prepulse inhibition (PPI) of the acoustic startle reflex (ASR) and usually referred to as GPIAS. As it does not require conditioning it became the method of choice to study neuroplastic phenomena associated with the development of tinnitus.

**Objective:** It is still controversial if GPIAS is really appropriate for tinnitus screening, as the hypothesis that a tinnitus percept impairs the gap detection ability (“filling-in interpretation” is still questioned. Furthermore, a wide range of criteria for positive tinnitus detection in GPIAS have been used across different laboratories and there still is no consensus on a best practice for statistical evaluation of GPIAS results. Current approaches are often based on simple averaging of measured PPI values and comparisons on a population level without the possibility to perform valid statistics on the level of the single animal.

**Methods:** A total number of 32 animals were measured using the standard GPIAS paradigm with varying number of measurement repetitions. Based on this data further statistical considerations were performed.

**Results:** We here present a new statistical approach to overcome the methodological limitations of GPIAS. In a first step we show that ASR amplitudes are not normally distributed. Next we estimate the distribution of the measured PPI values by exploiting the full combinatorial power of all measured ASR amplitudes. We demonstrate that the amplitude ratios (1-PPI) are approximately lognormally distributed, allowing for parametrical testing of the logarithmized values and present a new statistical approach allowing for a valid and reliable statistical assessment of PPI changes in GPIAS.

**Conclusion:** Based on our statistical approach we recommend using a constant criterion, which does not systematically depend on the number of measurement repetitions, in order to divide animals into a tinnitus and a non-tinnitus group. In particular, we recommend using a constant threshold based on the effect size as criterion, as the effect size, in contrast to the *p*-value, does not systematically depend on the number of measurement repetitions.

## Introduction

In western societies up to 15% of the general population suffer from subjective tinnitus (Heller, 2003), the perception of a sound in the absence of any acoustic stimulus. Despite this high prevalence and the tinnitus-associated distress of affected patients, which in severe cases may experience insomnia, psychological disorders like depression, the inability to work, or even commit suicide (Coles, 1984; Lewis et al., 1994; Langguth et al., 2011), there still is no effective cure for the condition, because all tinnitus research faces one central problem: Whereas the existence of a tinnitus percept can unequivocally be determined in human patients (one can simply ask them; cf. e.g., Pantev et al., 2012; Elgoyhen et al., 2015; Husain, 2016; Leaver et al., 2016), this is only unsatisfactorily possible in animal models for tinnitus (Von Der Behrens, 2014; Zhang et al., 2014; Galazyuk and Hebert, 2015; Brozoski and Bauer, 2016). On the other hand, the exact mechanisms within the auditory system that lead to the development of tinnitus are still unknown and hard to identify, since invasive neurophysiological methods that are essential for such research are only available in animal models but not in humans. Therefore, we still lack a mechanistic understanding of the tinnitus phenomenon which would indeed be crucial for the development of an effective cure. Accordingly, current tinnitus therapies mainly aim to help patients to cope with the condition rather than to cure it (e.g., Goebel et al., 1999; Zachriat and Kröner-Herwig, 2004; Westin et al., 2011). Consequently, what is needed most in tinnitus research is a reliable animal model suited to unravel the neurophysiological mechanisms of tinnitus development.

Currently, a number of different mechanistic models for the development of tinnitus do exist. To date, these models are mainly based on animal research (despite the above described fundamental problem of tinnitus research), are still only able to explain a subset of tinnitus phenomena like tonal tinnitus, and in addition, are discussed controversially (Gerken, 1996; Eggermont, 2003; Eggermont and Roberts, 2004; Engineer et al., 2011; Knipper et al., 2011; Schaette and Mcalpine, 2011; Wang et al., 2011; Yang et al., 2011; Ahlf et al., 2012; Ruttiger et al., 2013; Tziridis et al., 2015; Krauss et al., 2016b).

Originally, the assessment of tinnitus in animal models was based on some kind of conditioning where the animal learned to distinguish between conditions of sound vs. silence (Jastreboff et al., 1988a,b; Heffner and Harrington, 2002; Ruttiger et al., 2003). After training and induction of tinnitus (either by salicylate or noise trauma) the animals were expected to show sound-related behavior during the silence condition, and such behavior would then be considered indicative for the existence of a tinnitus percept. A major drawback of all conditioning approaches for tinnitus research is that any conditioning paradigm itself would trigger neuroplastic changes in auditory processing (Weinberger, 1993; Ohl et al., 2001; Ohl and Scheich, 2005) that potentially would interfere with neuroplastic phenomena that are related to tinnitus development. Hence, for any study that aims to unravel the neurophysiological mechanisms that underlie the development of tinnitus, conditioning paradigms for the assessment of tinnitus could lead to misinterpretations as it could be difficult to distinguish between learning induced and tinnitus induced neuroplastic changes in the auditory system (Norena et al., 2010).

Turner et al. (2006) proposed a new model for tinnitus assessment in animals that was based on gap-prepulse inhibition of the acoustic startle reflex (GPIAS). The ASR is a reflex to a loud acoustic stimulus in animals (Koch, 1999) and humans (e.g., Fournier and Hébert, 2016) and can be reduced by the perception of a pre-stimulus—here a gap in a continuous noise background. The reflex amplitude remains unchanged if the pre-stimulus is not perceived, and it is gradually decreased with the increase of the strength of the perception. Under the assumption that a possible tinnitus percept may fill the gap and thereby reduces the PPI of the ASR, it has even been tried to probe different frequency ranges (by using band-pass noise of different spectra as background) and identify the possible pitch range of the animals' tinnitus percept (Turner et al., 2006; Yang et al., 2007; Nowotny et al., 2011; Ahlf et al., 2012; Turner and Larsen, 2012; Tziridis et al., 2014, 2015; Liberman et al., 2015).

This new approach enjoys increasing popularity among the community of animal tinnitus researchers as it is much less time consuming than the aforementioned conditioning paradigms and seemingly simple (cf. Galazyuk and Hebert, 2015). Furthermore, the fact that it requires no conditioning prior to tinnitus testing, no conditioning-related plasticity is induced. Therefore, GPIAS seems well suited for studies that investigate mechanisms of tinnitus development.

Nevertheless, despite these obvious advantages it is still controversial if the method in general is appropriate for tinnitus screening, as the “filling-in” interpretation has been questioned (Campolo et al., 2013; Radziwon et al., 2015). Furthermore, a wide range of criteria for positive tinnitus detection have been used across different laboratories and there still is no consensus on a “best practice” for statistical evaluation of GPIAS results, as it exists for other behavioral paradigms (cf. Hinkle et al., 2003). Current approaches are often based on simple averaging of measured PPI values and comparisons on a population level without the possibility to perform valid statistics on the level of the single animal.

In this study we propose a straight forward, statistical stringent approach that could be used to harmonize and standardize GPIAS data analysis in future tinnitus research.

## Methods

### Animals and ethical statement

Mongolian gerbils (*Meriones unguiculatus*) were housed in standard animal racks (Bio A.S. Vent Light, Ehret Labor- und Pharmatechnik, Emmendingen, Germany) in groups of 2–3 animals per cage with free access to water and food at 20–24°C room temperature under 12/12 h dark/light cycle. The use and care of animals was approved by the state of Bavaria (Regierungspräsidium Mittelfranken, Ansbach, Germany, No. 54-2532.1-02/13). A total of 32 male gerbils aged 10–12 weeks were purchased from Janvier Laboratories Inc. and used in this study after acclimatization in our animal facility.

### Acoustic trauma

The pure tone acoustic trauma for tinnitus induction is applied under deep ketamine xylazine anesthesia as described in detail earlier (Ahlf et al., 2012; Walter et al., 2012; Tziridis et al., 2014, 2015; Krauss et al., 2016a). In a nutshell, the anesthetized animals were placed on a regulated heating pad with a temperature of 37°C central in front of a Loudspeaker (Canton Plus X Series 2; Canton, Weilrod, Germany). Using a signal generator (hp 33120A, HP, Böblingen, Germany) connected to an audio amplifier (Amp 75, Thomas Wulf, Frankfurt, Germany), a 2 kHz pure tone was presented at a sound pressure level of 115 dB SPL for 75 min.

### PPI of ASR measurements

For ASR measurements, as described earlier (e.g., Ahlf et al., 2012; Tziridis et al., 2012), animals were placed in a transparent acrylic tube (length 10 cm, inner diameter 4.3 cm) which was positioned at a distance of 10 cm in front of a loudspeaker (Canton Plus X Series 2), on a low-vibration table (TMC, Peabody, MA, USA). The whole setup was placed in an acoustic chamber (Industrial Acoustics Company GmbH, Niederkrüchten, Germany). The startle response was measured by a piezo force sensor (Honeywell FSG15N1A; sensitivity 0.24 mV/g; null shift at 25°C is ±1 mV; force range 0–1,500 g) attached underneath the tube. The front end of the tube was closed with a stainless steel grate (wire mesh, width 0.5 mm) allowing for acoustic stimulation with no detectable distortion within the used stimulation range of 250–8,000 Hz (signal-to-noise ratio at least 70 dB). Sound pressure level was calibrated using a condenser microphone (B and K Type 4190) via a preamplifier (B and K Type 2669) and measuring amplifier (B and K Type 2610). Stimulus generation and data acquisition was performed using custom-made programs (Matlab 2008, MathWorks, Natick, MA, USA). As startle amplitudes tend to be higher for the first few trials, five startle stimuli were presented before the beginning of each measurement to rule out strong habituation effects (Turner et al., 2006; Valsamis and Schmid, 2011).

The standard gap-startle protocol to measure behavioral correlates of tinnitus in rodents consists of several trials using a 20 ms long 115 dB SPL loud noise burst as startle stimulus presented in a continuous background band pass noise with a spectral width of half an octave centered on a given frequency. In half of the trials the “no-gap” condition, i.e., without any silent period within the background noise, was presented. In the other half of the trials—the “gap” condition—the band pass noise was interrupted by a 50 ms interval of silence, presented 100 ms before the startle stimulus. The stimulation was chosen according to the protocol used by Turner et al. (2006), but the stimulus frequency range was adapted to our animal model (cf. below). The response of the animals to the startle pulse was measured with the piezo force sensor described above.

Invalid trials (trials where the animal moved before the startle stimulus) were discarded by thresholding of the signal in the time interval 550 ms before the startle stimulus. The threshold was set to 0.5 mV. As the signal is superposed by high frequency measurement noise a low pass filter (butterworth 6th order, cutoff: 40 Hz was applied). The complete procedure is explained in detail in Supplementary Figure 1.

In a first protocol, we presented 200 trials with and 200 trials without a gap in a background noise centered at 2,000 Hz (± half an octave) to evaluate the distributions of the different responses of the animals to the two different stimulus conditions (in depth analysis of habituation effects is shown in Supplementary Figure 2). In other words all together 400 trials were presented to each animal (two stimulus conditions and 200 repetitions of each stimulus). In a second protocol we analyzed frequency dependent effects of the background noise as used in the standard protocol (cf. Turner et al., 2006): Here, only 15 trials with and without gap for each of 9 different center frequencies were presented (center frequencies: 500, 707, 1,000, 1,414, 2,000, 2,828, 4,000, 5,657, 8,000 Hz, all together 270 stimuli were presented). (For tinnitus testing, this protocol was measured before and after a pure tone acoustic trauma.) In both protocols the inter-stimulus intervals were randomized (10 ± 2 s) to exclude any possible adaptation or habituation of the animals to fixed time intervals (Joober et al., 2002; Ahlf et al., 2012; Krauss et al., 2016a,b).

Typically the startle reflex amplitude (A) is defined as the peak-to-peak amplitude of the reflexive response of the animal. According to Joober et al. (2002) and Jovanovic et al. (2004), the prepulse inhibition is defined as 1 minus the amplitude ratio of A_gap_ vs. A_nogap_:

(1) $$\begin{array}{r} PPI=1-\frac{A_{gap}}{A_{nogap}} \end{array}$$

where *A*_*gap*_ and *A*_*nogap*_ are the peak-to-peak response amplitudes for the gap and no-gap condition, respectively. Hence, the PPI value is always from the interval]−∞,1].

### Evaluation and statistics

The complete evaluation software including the applied statistical tests is written in Python 2.7 using the Pylab, Numpy and SciPy library, for scientific research (Hunter, 2007; Oliphant, 2007; Millman and Aivazis, 2011; Walt et al., 2011). All calculations were performed on a standard desktop PC. The statistical distributions were fitted using a maximum likelihood estimator provided by the stats library included in SciPy. Bootstrapped data sets were drawn using a self-written Python program based on the Numpy (random) library (Walt et al., 2011).

## Results

### Distribution of ASR peak-to-peak amplitudes

For any proper selection of statistical tests to be applied to a certain data set, knowledge about the distribution of values within the data set is crucial. Therefore, to obtain a valuable estimation of the distributions of startle reflex amplitudes and the PPI values, data from *n* = 6 animals were collected, with 200 gap and 200 no-gap condition measurement repetitions each. The stimulation paradigm for these measurements was a narrow band noise centered around 2 kHz with a spectral width of half an octave (cf. section Methods).

**Figure 1:**
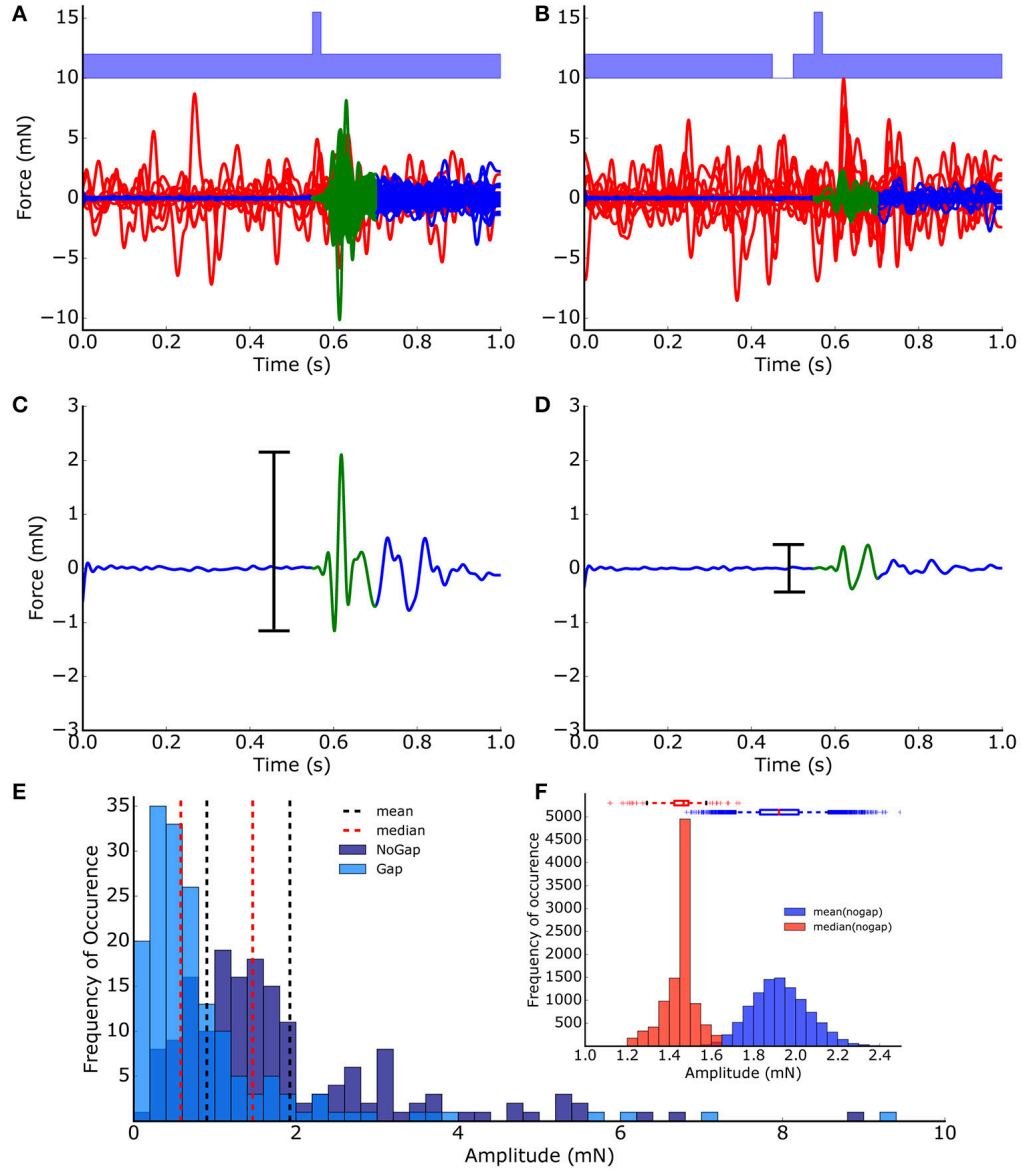
ASR peak-to-peak amplitude extraction and distribution. Startle reflexes (*N* = 200) of one animal from no-gap **(A)** and gap **(B)** condition measurements (light blue, band-noise, 2 kHz). Valid trials (blue/green lines) and invalid trials (red lines) were classified automatically based on a low-pass filtering and threshold procedure. **(C,D)** The peak-to-peak-amplitudes (black bars) of the 150 ms time intervals directly after the stimulus (green) was used as a measure of the strength of the startle reflex (startle reflex amplitude). **(E)** Frequency distribution of startle reflex amplitudes. **(F)** Distribution of means (blue) and medians (red) calculated from 10,000 bootstrapped data sets (no-gap ASR amplitudes, boxes: quartiles, whisker: 5–95% quantiles); The bootstrap procedure provides evidence that the median is the more robust measure for the startle amplitudes compared to the mean.

For processing the raw data, a fully automated procedure based on a MATLAB program has been applied. This fully automated evaluation of the startle reflex amplitudes provides the advantage that the evaluation is not influenced by any subjective bias. The program applies a low-pass (Butterworth, 6th order) filter with a cutoff frequency of 40 Hz to remove any high frequency background noise. The cutoff frequency was chosen not to distort the startle reflex amplitudes (cf. Supplementary Figure 1). Invalid trials, i.e., trials where the animal moved during the 550 ms time interval before the startle stimulus, were detected using an empirically determined threshold criterion (force > 0.2 mN = 0.5 mV) and discarded from further analysis (Figures 1A,B). The peak-to-peak amplitudes of the valid ASRs were calculated from the 150 ms time interval starting at stimulus onset (Figures 1C,D).

The distributions of the ASR amplitudes for gap (*A*_*gap*_) stimuli are compared to those of the no-gap condition (*A*_*nogap*_), as this is critical for any statistical testing of PPI changes. Obviously, the peak-to-peak ASR amplitudes (Figure 1E) were not Gaussian-like distributed, indicating that standard parametric testing procedures, (such as *t*-testing) cannot be applied to ASR amplitudes, ratios of ASR amplitudes or PPI values. Or in other words, the mean of the peak-to-peak ASR amplitude is highly influenced by outliers and therefore, may not be considered a good statistical measure. This is further demonstrated with 10,000 bootstrapped data sets drawn from the measured no-gap ASR amplitudes (of the shown animal) as shown in Figure 1F, where the distributions of the medians as well as the means are given. The accompanying boxplots provide evidence that the variance of the means is significantly higher than the variance of the medians. Taken together, any statistical analysis of raw ASR data must be based on non-parametrical testing. For example, Mann-Whitney U-statistics could be applied to test if presentation of a gap in noise before the startle stimulus led to a significant PPI (as evident in Figure 1C vs. Figure 1D: *p* < 0.001) which represents the basis for GPIAS behavioral testing for tinnitus assessment.

### Distribution of PPI values

As demonstrated in the previous section, ASR amplitudes are broadly distributed and skewed. Therefore, comparing the ASR amplitudes with and without gap cannot simply be achieved by using the mean and standard deviation, but the whole distribution has to be taken into account. From our dataset (200 measurement repetitions at 2 kHz band noise gap and no-gap, respectively), the ratio of all combinations of gap and no-gap ASR amplitudes was calculated (cf. Supplementary Figures 3, 4). The full combinatorial number *N*_*PPI*_ of all possibly ASR amplitude combinations is given by:

(2) $$\begin{array}{r} N_{PPI}=N_{A_{gap}}\;\cdot \;N_{A_{nogap}} \end{array}$$

Hence, if all measurements are valid, a maximal number of 40,000 PPI values can be calculated. The calculation of all combinatorial PPI values is a valid estimator for the distribution of the compound variable (combination of several variables, Poe et al., 2005).

**Figure 2:**
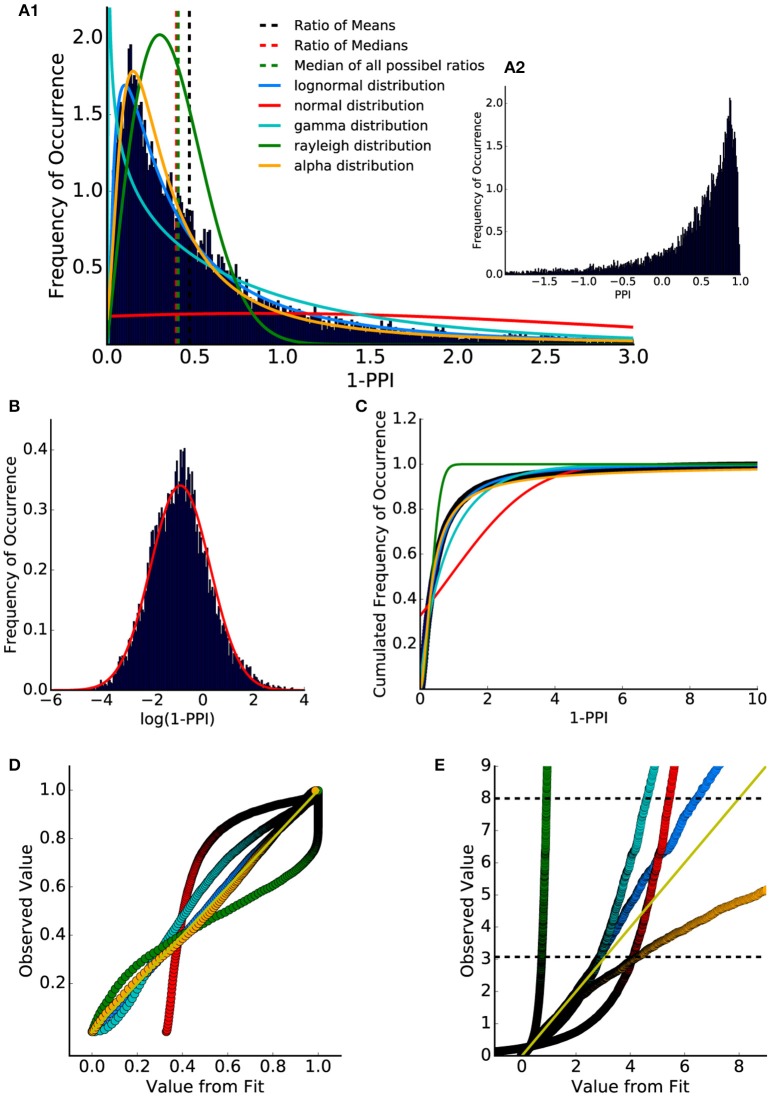
Distribution of ASR amplitude ratios and PPI values. **(A1)** Ratio between all combinations of gap and no-gap ASR amplitudes (1-PPI). Data were fitted with lognormal (solid blue), gamma (solid cyan), Rayleigh (solid green), and alpha (solid orange) distribution (normed probability densities). Ratio of means of gap and no-gap ASR amplitudes (dashed black) differed from the ratio of medians of gap and no-gap ASR amplitudes (dashed red), which is similar to the median of the (full combinatorial) ratio distribution (dashed green). **(A2)** Distribution of PPI values (1-A_gap/A_no-gap). The histogram shows that the PPI values cover a wide codomain including a considerable number of negative values (]−∞,1]). **(B)** Histogram of the logarithmized (base e) ASR amplitude ratios. When plotted this way, the data are almost Gaussian-like distributed. **(C)** Cumulative distribution of the ASR amplitude ratios (dark blue dots) and cumulative distribution function of the fitted distributions: lognormal (blue) and Gaussian (red), gamma (cyan), Rayleigh (green) and alpha (orange). **(D)** p-p plot shows that the lognormal distribution describes the data best. However, the q-q plot **(E)** provides evidence that for percentiles higher than 95% (lower black dashed line) the lognormal distribution slightly differs from the measured values.

To compare the histogram of the PPI values to standard stochastic distributions, ASR amplitude ratios (= 1-PPI; cf. Equation 1) were used to shift the value range from]−∞, 1] (Figure 2A2) to [0, ∞[(Figure 2A1). Fitting different distributions to the data using a maximum likelihood estimator revealed that the lognormal distribution provides the highest likelihood and therefore estimates the true data distribution best. Consequently, the calculated logarithm of the ASR amplitude ratios was approximately normally distributed (Figure 2B). The finding that the ratio distribution can be approximated with a lognormal distribution has been reported for standard startle paradigms in humans and mice (Csomor et al., 2008). Additionally, the Akaike information criterion was used to quantify which distribution fits best. This criterion introduces a penalty for the number of used fit parameters preventing overfitting (Akaike, 1974; Saffron et al., 2006). This criterion also leads to the result that the lognormal distribution fits best to the observed data.

Therefore, although parametric statistics may not be applied to raw ASR amplitude ratio data, parametric statistics may be applied to logarithmized ASR amplitude ratio data.

To further examine the underlying statistical distribution, a quantile analysis was performed, allowing for the evaluation of how well a given distribution describes the data (q-q plot and p-p plot, Michael, 1983; Gan and Koehler, 1990; Holmgren, 1995). The cumulated distribution (integral function of probability density) of the fitted distributions and of the data (ratio histogram) were calculated (Figure 2C).

Figures 2D,E show so called p-p and q-q analysis respectively. These plots indicate how well the histogram could be described by the fitted distributions, with a perfect fit resulting in all supporting points lying on the identity line for p-p (Figure 2D) as well as for q-q plots (Figure 2E).

For q-q analysis the quantiles of the data are plotted as a function of the quantiles of the fitted distribution. The q-q analysis emphasizes the edges of the distributions. One scale invariant representation is the so called p-p plot (Holmgren, 1995) comparing the cumulated probabilities (cannot exceed 1). Thus, the upper limit of a p-p plot is always one. This q-q plot shows that the ratio distribution could be nicely described by a lognormal distribution up to the 95% percentile (further animals are shown in Supplementary Figure 5).

As the standard procedure for tinnitus detection in animal models is the analysis of the PPI decrease due to a treatment (in most cases an acoustic trauma, Bauer and Brozoski, 2001; Norena and Eggermont, 2003; Yang et al., 2011; Ahlf et al., 2012; Tziridis et al., 2015; Krauss et al., 2016b) the next section discusses possible measures for PPI change and valid inferential statistical tests.

### GPIAS statistics

As demonstrated above, the ASR amplitude ratios (1-PPI) can be described by lognormal distributions up to the 95% quantile, so that parametric statistics may be applied if data are logarithmized. To test if the ASR amplitude ratio distributions changed significantly post trauma relative to pre trauma conditions, the combined standard error of the logarithmized ASR amplitude ratios was calculated. The logarithmized ratio of the ASR amplitudes is given by the difference:

(3) $$\begin{array}{r} \log\left(\frac{A_{gap}}{A_{nogap}}\right)=\log\left(A_{gap}\right)-\log\left(A_{nogap}\right)\\ =L_{gap}-L_{nogap} \end{array}$$

**Figure 3:**
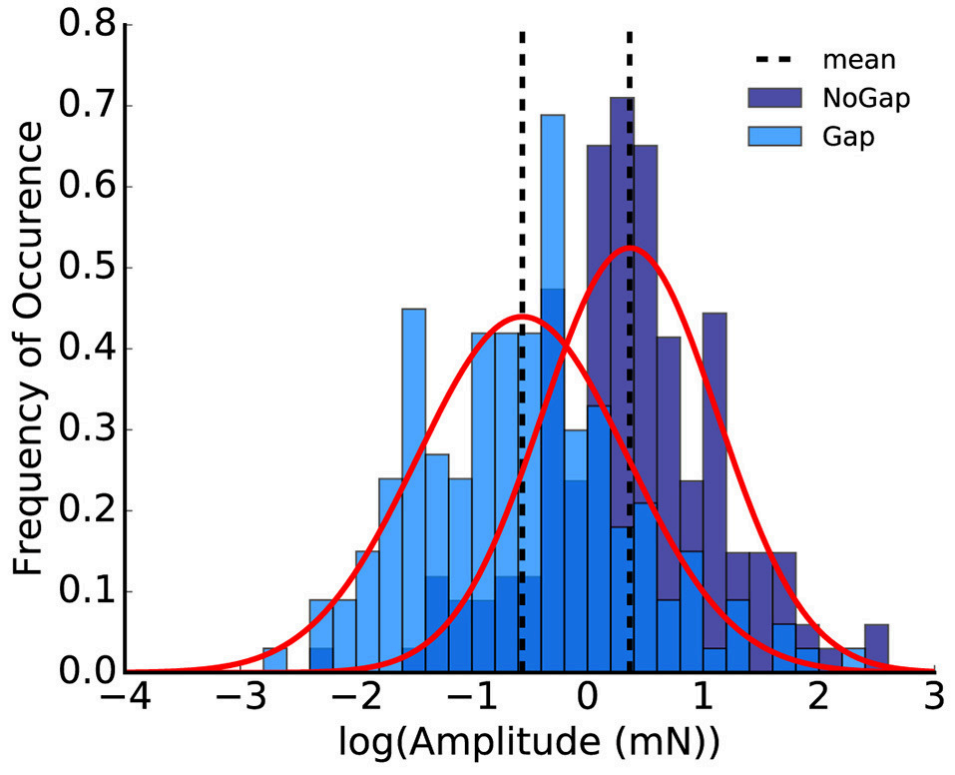
Histogram of the logarithmized peak-to-peak amplitudes; the plot shows the animal replot of the data from Figure 1E. Shapiro-Wilk-test for normality emphasizes that the logarithmized amplitudes are Gaussian-like distributed (*p* = 0.11 for no-gap and *p* = 0.21 for gap, normalized probability density).

As the logarithmized ASR amplitude ratios are Gaussian-like distributed, and the difference of two Gaussian-like distributed random variables is again Gaussian-like distributed (Eisenberg and Sullivan, 2008; Kersting and Wakolbinger, 2008), one may infer that also the logarithmized ASR amplitude values for both the gap and the no-gap condition could be Gaussian-like distributed (however it is not crucial that they are Gaussian distributed). That this is at least the case for our data set could be confirmed using the Shapiro-Wilk test (Shapiro and Wilk, 1965) (Figure 3, *p* > 0.1 for both the gap and the no-gap condition). The standard error for the distribution of the logarithmized ASR amplitude ratios can therefore be calculated using error propagation.

(4) $$\begin{array}{r} \Delta L_{ges}^{2}=\Delta L_{gap}^{2}+\Delta L_{nogap}^{2} \end{array}$$

($\Delta L_{ges}^{\;}$: standard error of means of the logarithmized ratios, $\Delta L_{gap}^{\;}$/$\Delta L_{nogap}^{\;}$: standard error of means of logarithmized gap and no-gap amplitudes)

Furthermore, the variance of a compound Gaussian-like distributed measure is simply the sum of the variances (Satterthwaite, 1941). The effective number of independent samples of the compound variable can be calculated using the variance and the standard error

(5) $$\begin{array}{r} n=\frac{Var\left(L_{ges}\right)}{{\Delta L_{ges}}^{2}} \end{array}$$

where *Var*(*L*_*ges*_) is the variance of the compound distribution and Δ*L*_*ges*_ the standard error of the compound distribution. However, this effective *n* is only an approximation and may also be replaced by a more conservative estimation (cf. Supplementary Figures 7D–F). The information on the standard error of the compound variable (logarithmized ratios) and an effective *n* makes it possible to test if the pure tone acoustic trauma leads to a significant change of the logarithmized ratios. In other words, the null hypothesis (H_0_) can be formulated as follows:

The logarithmized ratios before ($L_{ges}^{pre}$) and after ($L_{ges}^{post}$) the acoustic trauma arise from the same distribution. (Note that this test is done for each stimulus center frequency individually).

Calculating the values *Mean*(*L*_*ges*_) and $\Delta L_{ges}^{2}$ for pre- and post-trauma conditions allows calculating the T statistics for the comparison between pre- and post-trauma conditions:

(6) $$T=\;\frac{Mean\text{​}\left(L_{ges}^{pre}\right)-Mean\left(L_{ges}^{post}\right)}{\sqrt{\Delta L_{ges}^{pre^{2}}+}\Delta L_{ges}^{post^{2}}}$$

The mean of the logarithmized ratios ($Mean\left(L_{ges}^{pre/post}\right)$) is the difference of the means of the logarithmized gap and no-gap amplitudes. However, it can also be regarded as the mean of the full combinatorial difference of logarithmized gap and no-gap amplitudes. It can be shown that these two possibilities are equal (cf. Supplements, Equation 2).

Finally, using the T-statistics, a *p*-value can be calculated:

(7) $$\begin{array}{r} p=2\cdot \int_{-\infty }^{-|T|}Stud\left(t,df\left(n\right)\right)dt \end{array}$$

Where T refers to the test statistics, Stud to the students T-distribution and df(n) are the degrees of freedom (Monte Carlo simulation used to prove validity of statistics cf. simulation study Supplementary Figures 6, 7).

In summary, the paragraph shows how to calculate the *p*-value when comparing the logarithmized ASR-ratios before and after an acoustic trauma for one specified stimulus frequency (band noise center frequency) and one animal. The *p*-value is a measure for the probability that the null hypothesis (pure tone trauma has no effect on ASR amplitude ratios) is falsely rejected. In other words, this *p*-value indicates if the effect of a change of the average ratio of gap and no-gap amplitudes is by chance (sampling error) or if there exists a real effect. However, this measure only indirectly gives information on the size of the effect. Furthermore, it has to be considered that not significant (*p* > 0.05) does not mean that the trauma did not lead to any effect (e.g., development of a tinnitus percept) but that the sample size is too small to detect the effect or that there is no effect. The *p*-value makes no statement about the second order error.

### Effect size as a novel and normed measure for the PPI change (ΔPPI)

**Figure 4:**
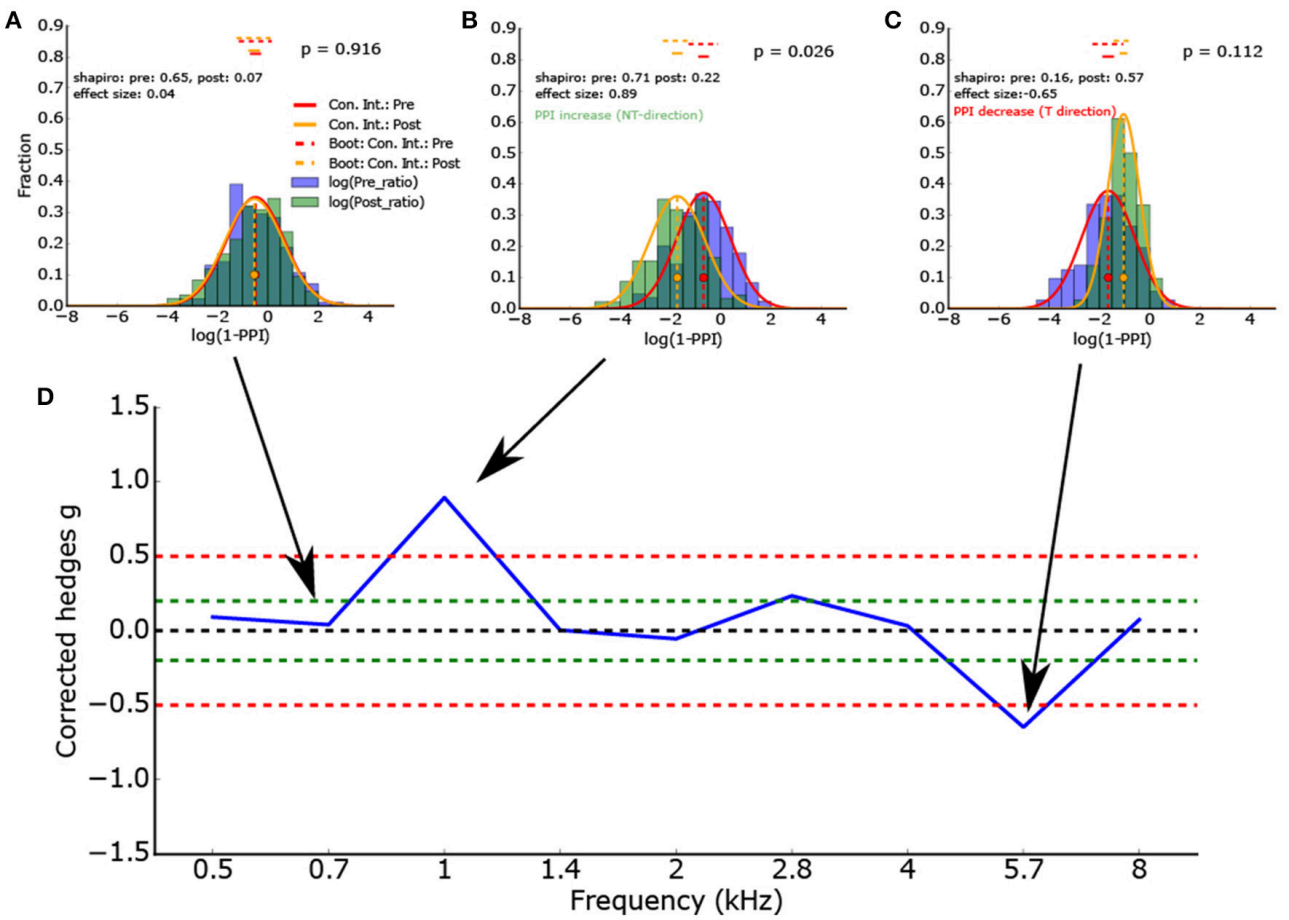
**(A–C)** Histogram of the logarithmized ratios of gap and no-gap amplitudes (full combinatorial) for different center frequencies (0.7, 1.0, and 5.7 kHz) of the band noise presented. As the ASR amplitude ratios are almost lognormal distributed, the logarithmized values are Gaussian-like distributed. Application of the Shapiro-Wilk test proves that the normal distribution is a valid description of the data. The red and orange vertical lines show the full combinatorial 95% confidence intervals. The dashed vertical line show the 2.5–97.5% quantile of the means calculated via bootstrapping. The size of the bootstrapped data sets was the minimum of the number of peak-to peak amplitudes of gap and no-gap measurement. In other words the bootstrapped data sets provide the upper limit of the variance of the determined means (the confidence intervals). **(D)** To determine the PPI change, the corrected effect size is used (n1, n2 same sample size used for statistics). To test if the distributions differ significantly, inferential statistics were applied. However, as the ASR amplitude ratios arise from all combinations of gap and no-gap amplitudes, it is possible that the number of independent ASR amplitude ratios is overestimated.

Furthermore, a novel measure for the ΔPPI (until now: ΔPPI = PPI_post_ - PPI_pre_) and hence for the tinnitus percept can be defined using the effect size (Figure 4D). This measure for the PPI change is normalized and not dependent on the dimension of the measured variables. This measure is based on two Gaussian distributions and represents the difference of the means in terms of standard deviations (Figures 4A–C).

As the sample sizes are not equal, the definition by Hedges (Zimmermann et al., 2005; Nakagawa and Cuthill, 2007; Hofmann and Smits, 2008) has to be used.

(8) $$\begin{array}{r} g=\left(Mean\left(L_{ges}^{pre}\right)-Mean\left(L_{ges}^{post}\right)\right)/s \end{array}$$

(9) $$\begin{array}{r} s=\sqrt{\frac{\left(n_{pre}-1\right)\cdot \;Var\left(L_{ges}^{pre}\right)+\left(n_{post}-1\right)\;\cdot \;Var\left(L_{ges}^{post}\right)}{\left(n_{pre}+n_{post}-2\right)}} \end{array}$$

(s: combined, weighted standard deviation, $L_{ges}^{pre/post}$: logarithmized ratios for pre and post condition, respectively, *n*_*pre*/*post*_ sample size same as for Welch-*t-*test, as variance estimator of $L_{ges}^{pre/post}$ the sum of the variences of logarithmized gap and no-gap amplitudes are used).

Furthermore, the effect size (Hedges g) can be corrected for small sample sizes (Hedges, 1982; Nakagawa and Cuthill, 2007):

(10) $$\begin{array}{r} g^{*}=\left(1-\frac{3}{4\cdot \left(n_{pre}+n_{post}\right)-9}\right)\cdot g \end{array}$$

In the following the effect size is used as synonym for the sample size-corrected version of Hedges g (g^*^).

Figure 4D gives the effect size (corrected Hedges g) of one exemplary animal as a function of the frequency spectrum (center frequency) of the band noise presented. This animal showed a clear effect (ASR amplitude ratio decrease, PPI increase) at a center frequency of 1 kHz and a PPI decrease at a frequency of 5.7 kHz, indicating a potential tinnitus percept there.

**Figure 5:**
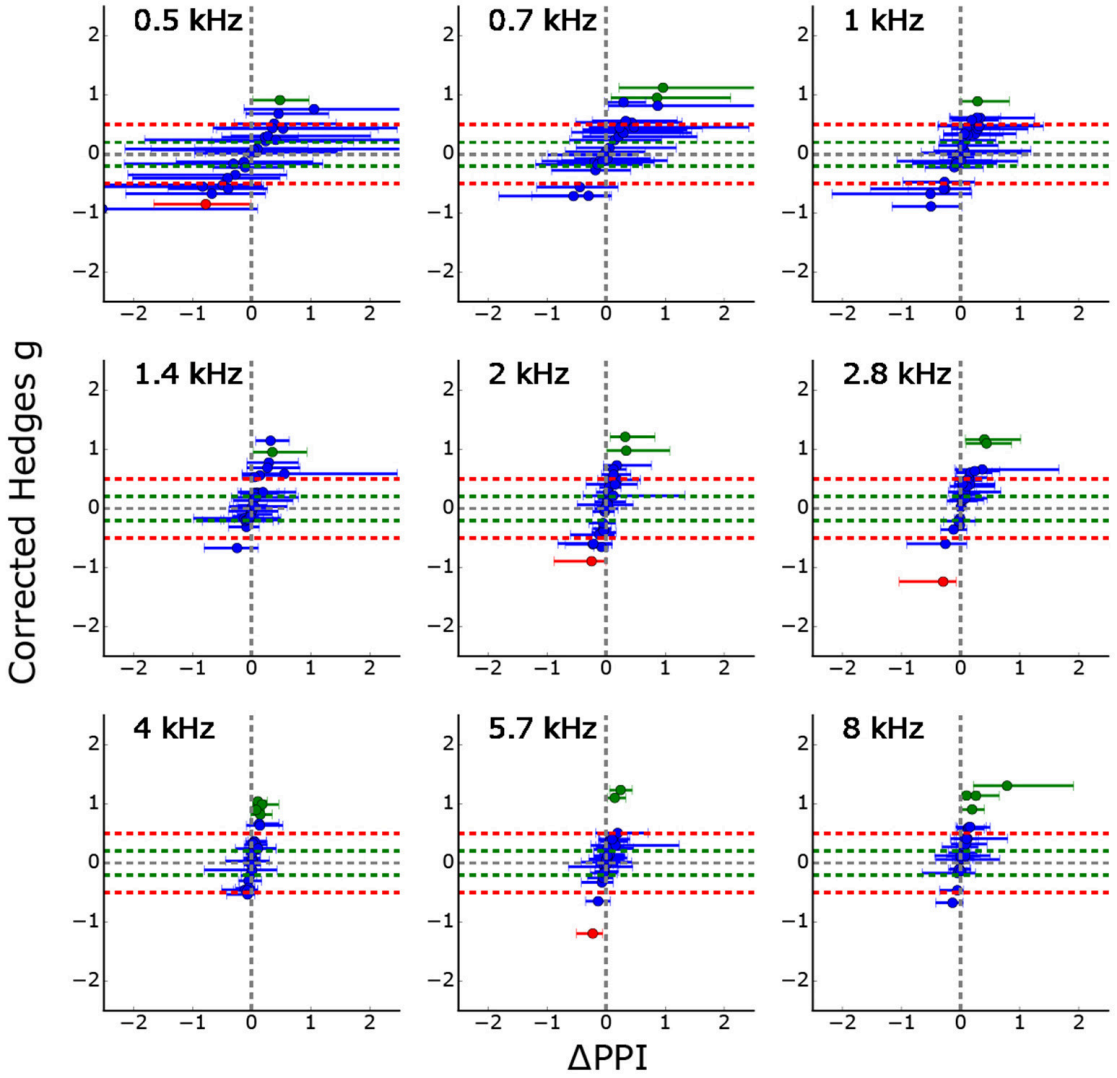
Corrected effect size as a function of the median ΔPPIs for all 26 animals (error bars: quartiles). Significant PPI decrease (based on Welch-Test cf. section GPIAS statistics, *p* < 0.05) is marked in red whereas significant PPI increase is marked in green. Trivially, the effect size and the median ΔPPI are highly correlated, but the effect size is additionally normed by the standard deviation of the logarithmized ASR amplitude ratio distributions. The figure shows that only three PPI changes become significantly smaller (red) for all 26 animals and 9 stimulus frequencies each.

Figure 5 summarizes the GPIAS results for all 26 animals to which the standard tinnitus paradigm was applied. It shows the effects size as a function of the median of the classical ΔPPI (calculated from the full combinatorial of all pre and post PPI values, error bars: quartiles, complete procedure of full combinatorial calculation shown in Supplements, cf. Supplementary Figures 3, 4), significant PPI changes are either colored in red (PPI decrease) or green (PPI increase). Trivially, effect size and ΔPPI are highly correlated, but the effect size is normed by the standard deviations of the logarithmized ASR amplitude ratio distributions. Using this new statistical criterion, only three animals show a significant PPI decrease indicating that the number of measured trials for the standard Turner paradigm might be too low to see small effects.

## Discussion

To date, there are no proper, universally accepted and used statistics for the determination of a significant change of the PPI as an indicator for a tinnitus percept in animals. With this study we attempted to provide such a statistical approach for variance estimation of PPIs and for reliably testing if PPI changes in a GPIAS paradigm, e.g., after trauma, are significant. The method is robust and does not require any removal of outliers, which otherwise is a common procedure (e.g., Longenecker and Galazyuk, 2011), and therefore can be applied fully automated.

The basis of that analysis is that the ASR amplitude ratios (1-PPI) of gap and no-gap ASR amplitudes are lognormally distributed for percentiles lower than 95%, estimated by calculating the full combinatorial of the gap and no-gap ASR amplitudes and q-q-analysis and p-p-analysis. In addition, the Shapiro-Wilk-test for normality provides evidence that the logarithmized ASR amplitude ratios are well described by a normal distribution. Hence, the effect size can be used as a normalized measure for the PPI change. Finally, the Welch-*T*-test, used on the propagated error, provides a measure for the significance value of that change.

In contrast to the statistics proposed here, earlier evaluation procedures calculated PPI values by simply combining the averaged gap and no-gap ASR amplitudes (Lehmann et al., 2000; Joober et al., 2002; Jovanovic et al., 2004; Wolff and Bilkey, 2010) and therefore the information about measurement uncertainties (variance) was removed. As a result of such procedures, it is impossible to provide information on the variance of the PPI values as well as the *p*-value of a possible PPI change. Some approaches tried to overcome these limitations by averaging the ASR amplitudes during the gap condition and dividing it by all ASR amplitudes during the no-gap condition (Longenecker and Galazyuk, 2011; Tziridis et al., 2012). However, thereby the *t*-test was erroneously used on the averaged variables, which are not normally distributed and therefore, may not be tested parametrically. Furthermore, there is no clear rule which ASR amplitudes (gap or no-gap) should be averaged and consequently the methods are ambiguous. The results of dividing all gap amplitudes by the averaged no-gap amplitudes obviously leads to different results than dividing the averaged gap amplitude by all no-gap amplitudes.

Additionally, it should be noted that averaging of one variable amplitude (gap or no-gap) leads to an underestimation of the error of the compound variable (ratio) and therefore applying inferential statistics to this data leads to an overestimation of the *p*-value.

All these calculations are usually performed to obtain information about the existence of a possible tinnitus percept in the animal tested. In tinnitus research, animals are often divided into a tinnitus group (T; based on significant PPI decrease in the GPIAS paradigm) and a second, no-tinnitus group (NT) containing the animals showing no significant PPI decrease after an acoustic trauma. The criterion for T animals is usually that a significant PPI decrease (Ahlf et al., 2012; Tziridis et al., 2015; Krauss et al., 2016a) at least in one specific frequency can be observed. Thereby the significance level α has to be corrected as one false positive value, out of all tested frequencies would lead to a false positive T-animal status. By using the Bonferroni-correction, the significance level α has to be adapted to the number of measured stimulus frequencies by dividing the value by the number of measured frequencies, but this has not been done in many studies using GPIAS. In our approach proposed here, the PPI change at one stimulus frequency is significant if the calculated significance level is lower than α = 0.05/9 = 0.0056. This correction is only used to identify T-animals, if the stimulation frequencies are treated individually and the α-value is set to 0.05. A reduction of the α-value for single frequencies would lead to a higher second order error. Additionally, by performing this analysis it has to be considered that classification as NT does not mean that the animals definitely have no tinnitus, but only that no significant PPI change for any tested stimulus frequency could be determined.

Despite these statistical considerations further criteria for the separation of T and NT animals should be taken into account.

In classical GPIAS approaches, as the *p*-value is highly dependent on the number of measured trials (measurement repetitions), the fraction of T animals will rise systematically with increasing measurement repetitions (number of repetitions of the same stimulus to better estimate the underlying distribution of the startle amplitudes).

Hence, altering of the number of applied stimulus repetitions leads to a systematic shift of the number of T classified animals (cf. Supplements: “Classification of T animals: significance criterion compared to effect size threshold” and Supplementary Figure 8).

One modern method to compare the distributions of test statistics of two groups, even if these distributions are not Gaussian, would be Bayesian statistics (Kruschke, 2013). However, even this approach does not solve the problem of systematically rising number of T classified animals for increasing number of measurement repetitions. We therefore refrained from elaborating it here.

Therefore, we here propose to choose a criterion independent from the number of trials, such as the effect size. Trivially, an increase of measurement repetitions will then lead to a more exact estimation of the effect size (cf. Supplementary Figure 8). However, no systematic deviation of the effect size depending on the number of measurement repetitions is observable. In other words, we believe that a separation of T and NT animals based on the effect size of PPI change in GPIAS represents a more reliable approach for tinnitus assessment in animals than the commonly used significance criteria.

In summary, the study demonstrates that ASR amplitudes for single animals are not Gaussian-like distributed. Furthermore, the ratios of ASR amplitudes during gap and no-gap conditions are well (although not perfectly) described by lognormal distributions. Based on this insight it is possible to estimate a *p*-value specifying if any observed PPI change after an acoustic trauma is significant. Alternatively, it is possible to calculate a normed measure, the effect size, which can be used to divide animals in T and NT animals as it is not systematically influenced by the number of applied measurement repetitions but should saturate at a certain value (cf. Supplementary Figure 8).

## Author contributions

AS, HS, KT, and PK designed the study; AS performed the measurements and implemented the computer simulations; AS, RG, PK, CM developed and discussed the statistics; AS, HS, KT, RG wrote the manuscript.

### Conflict of interest statement

The authors declare that the research was conducted in the absence of any commercial or financial relationships that could be construed as a potential conflict of interest.
