# Supplementary material for: A New Statistical Approach for the Evaluation of Gap-prepulse Inhibition of the Acoustic Startle Reflex (GPIAS) for Tinnitus Assessment
Source: Front Behav Neurosci. 2017 Oct 18;11:198. doi: 10.3389/fnbeh.2017.00198 (PMC5651238; doi:10.3389/fnbeh.2017.00198)
Supplement: Supplementary file 1 [file DataSheet1.PDF]

Supplements for A new statistical approach for the evaluation of gap-prepulse inhibition of the acoustic startle reflex (GPIAS) for tinnitus assessment

Achim Schilling, Patrick Krauss, Richard Gerum, Claus Metzner, Konstantin Tziridis, and Holger Schulze

Contents

1 Processing of the raw data **2**

2 Habituation effects on the PPI of the ASR **3**

3 Calculation of the full combinatorial **4**

4 Further examples for ratio distributions **6**

5 Simulation study **6**

6 Classification of T (tinnitus) animals: significance criterion compared to effect size threshold **11**

7 References **12**

1 Processing of the raw data

To discard invalid trials, i.e. trials where the animal moved before the startle stimulus, a lowpass filtering procedure was applied to the raw data measured via the piezo force sensor (Honeywell FSG15N1A; sensitivity 0.24 mV/g; null shift at 25 °C is ± 1 mV; force range 0 to 1500 g). The high frequency measurement noise is removed by the application of a 6th order Butterworth lowpass filter with a cutoff frequency of 40 Hz. Suppl.Fig. 1 shows the raw signal (red) measured via the data acquisition card and the resulting signal after lowpass filtering (blue). The figure shows that the application of the lowpass filter has only marginal effect on the amplitude or the latencies. This filtering procedure enables for the deletion of invalid trials

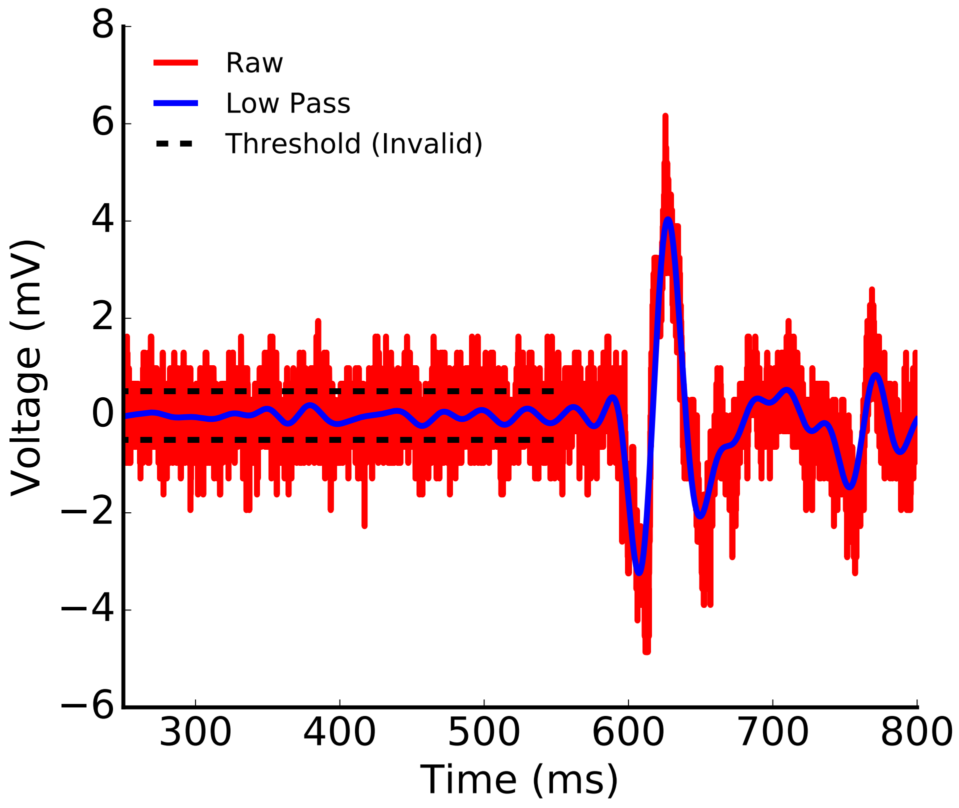

Supplementary Figure 1: **Low pass filtering of the raw data for reduction of measurement noise and thresholding for invalid trial deletion**
The plot shows the raw data measured via piezo sensor connected to the data acquisition card (red). The blue curve shows the same data after lowpass filtering. Invalid trials were discarded by simple thresholding. If the blue curve exceeds on of the black dashed lines, the trial was discarded.

(movement of the animal in the time interval of 550 ms before startle pulse onset) by simply setting a threshold (± 0.5 mV, black dashed line).

2 Habituation effects on the PPI of the ASR

Habituation effects during ASR measurement are always an issue (cf. Lauer et al., 2017). Suppl. Fig. 2 shows the progression of the ASR amplitudes as a function of startle trials (altogether 400 trials, 4h) for the pre-trauma measurements (6 animals). During the complete measurement procedure the startle amplitudes monotonically decrease. However, the fractional decrease of ASR amplitudes during gap and no-gap conditions is not constant. Consequently, also the PPI values (red) decrease and therefore a removal of the habituation effects by pairwise application and analysis of gap and no-gap conditions is in principal not possible. Therefore, the estimation of the distribution of PPI values by the full combinatorial calculation of all ASR amplitude pairs during gap and no-gap conditions as proposed here seems to be the most sophisticated approach.

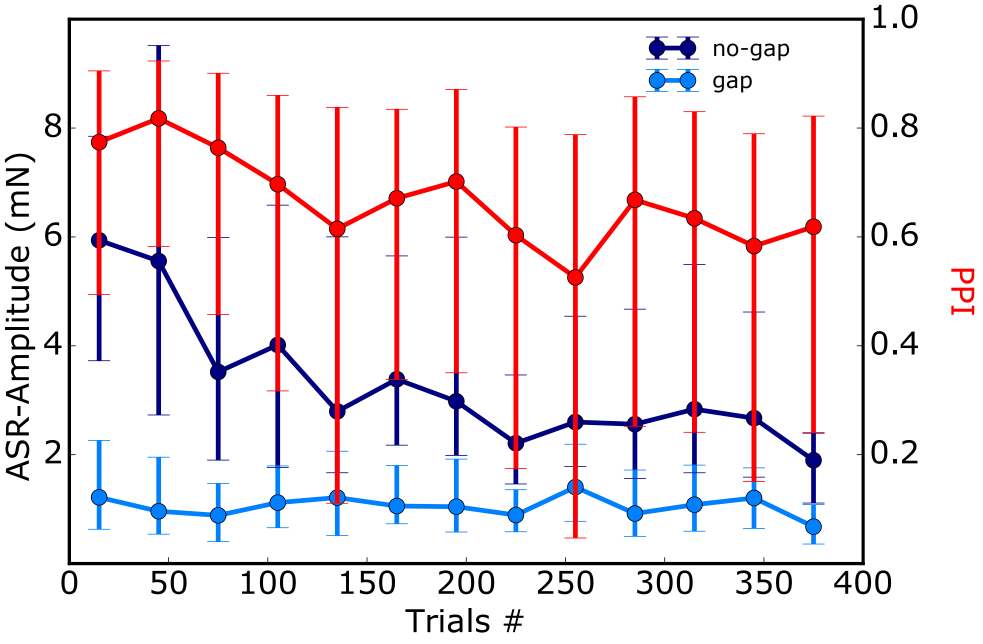

Supplementary Figure 2: **Habituation effects during startle measurements**
ASR amplitudes to startle pulses with and without pre stimulus (cyan, blue, median ± quartiles) and corresponding PPI values (red, median ± quartiles) for the GPIAS paradigm (2 kHz, 200 trails gap and no-gap condition each, cf. sec. 3.1) as a function of the ASR trials. The data were binned (30 trials per bin). Invalid trials were not considered for binning, but counted for the trial number. The fractional ASR amplitude decrease was not constant (i.e. not the same slope) for ASR amplitudes during gap and no-gap conditions, and hence the PPI values decreased as a function of ASR trials. The PPI change was significant (Kruskal-Wallice-test: $p<0.001$, Mann-Whitney-U statistics (comparing last to first bin) $p<0.001$).

3 Calculation of the full combinatorial

Suppl. Fig. 3A to C explain the stepwise calculation of the full combinatorial of the ΔPPI shown in Fig. 5 of the main text. In Suppl. Fig. 4 the ΔPPI (calculated from full combinatorial) as a function of the ΔPPI calculated from simple ASR amplitude means is presented. It is obvious that the two measures for the ΔPPI are highly correlated, nevertheless, data points in quadrant II and IV (upper left, bottom right) also exist. These data points represent differently classified ΔPPIs, demonstrating that simple averaging of ASR amplitudes leads to qualitatively different classification of the animals in T or NT (e.g. ΔPPI<0 for full combinatorial or ΔPPI>0 for averaging approach). Additionally and in contrast to the averaging approach, the full combinatorial provides information about the variance of the ΔPPIs.

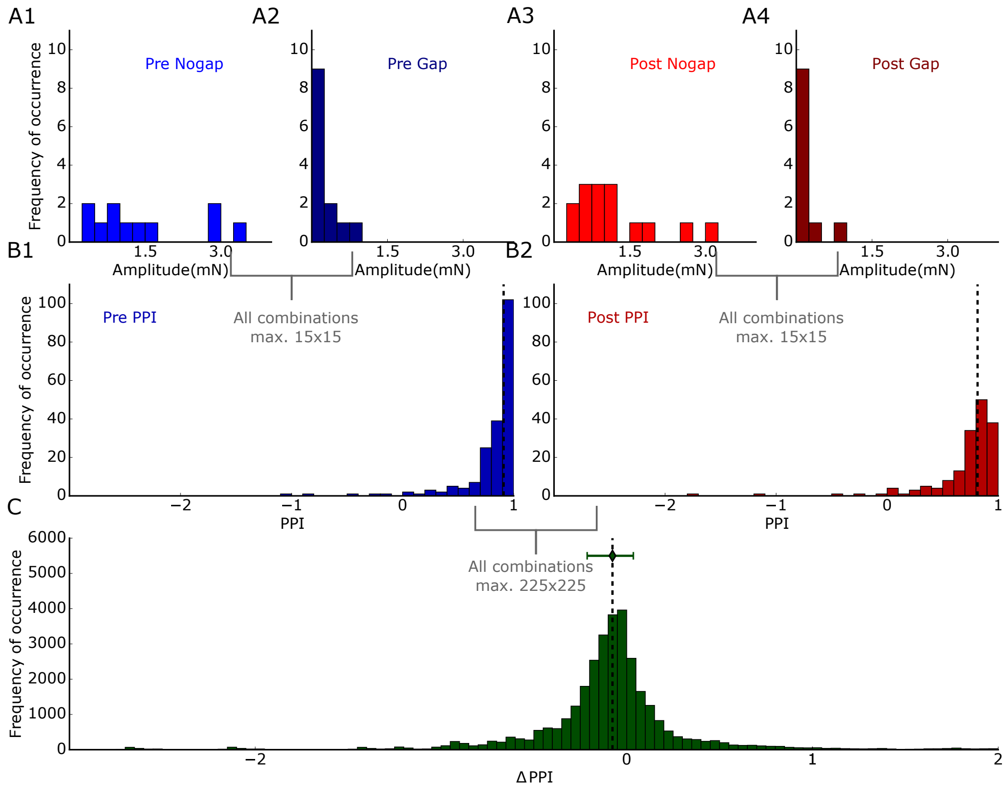

Supplementary Figure 3: **Calculation of the full combinatorial (ΔPPI)**
A1, A2, A3, A4: ASR amplitude distributions for one animal and one band noise stimulation with a center frequency of 2 kHz during four different conditions (pre- and post-trauma, gap and no-gap). The histograms show that the frequency of occurrence of high ASR amplitudes was decreased for the gap conditions (A2, A4) compared to no-gap conditions (A1, A3). All ASR amplitudes were used to calculate the distribution of the PPI values for the pre-trauma (B1) and the post-trauma (B2) condition. The median of the ΔPPI was calculated from all possible ΔPPIs (C, full combinatorial). The error bar marks the quartiles of the distribution.

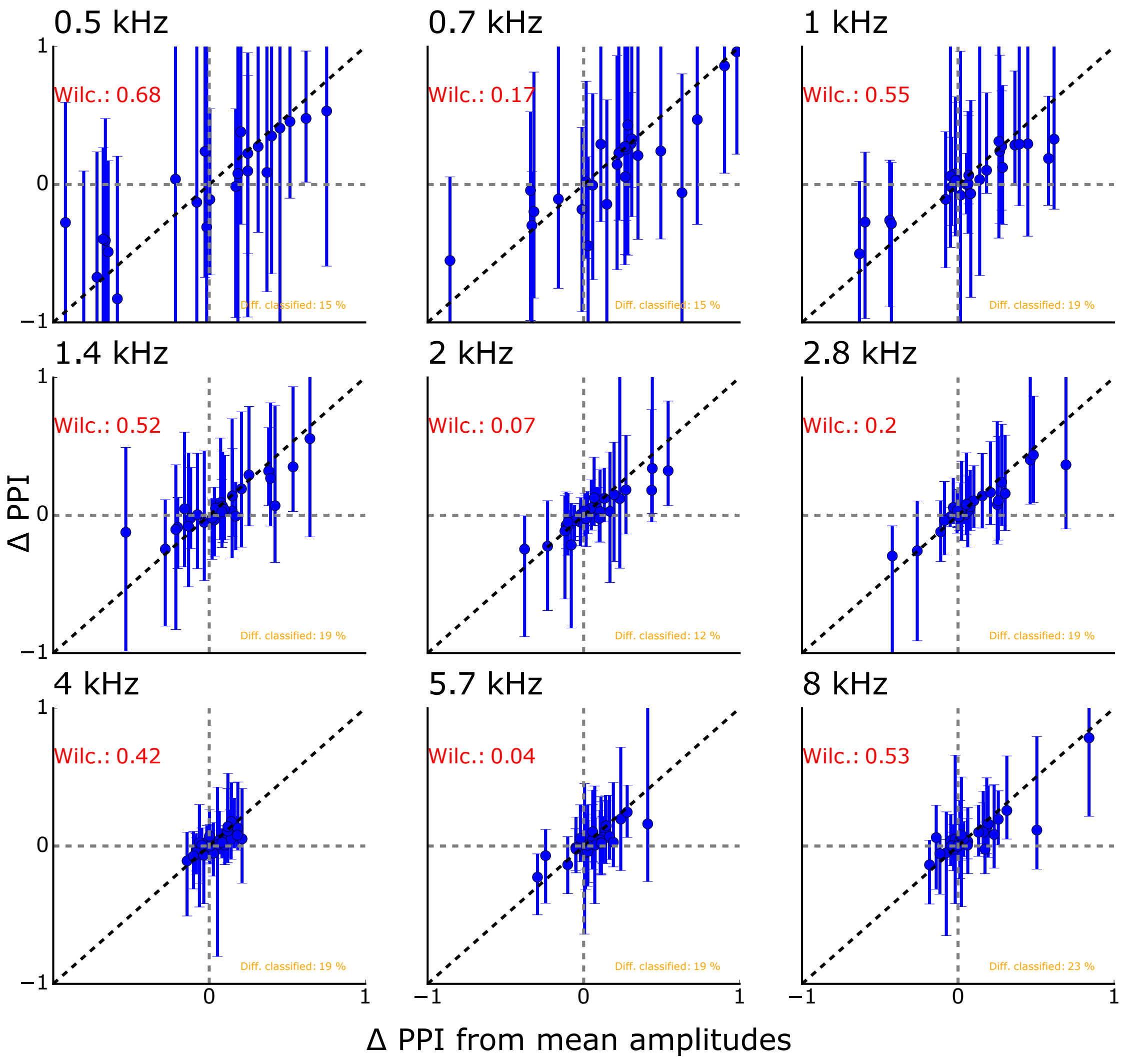

Supplementary Figure 4: **Comparison ΔPPI calculation methods.**
Comparing the standard method for ΔPPI calculation by taking the mean of ASR amplitudes during gap and no-gap conditions for PPI calculation (1-mean(gap)/mean(no-gap)) and the novel approach proposed here taking all ASR value combinations into account (Suppl. Fig. 3). The median values of the novel approach are almost equal to the mean values obtained by the old method. However, data points in quadrant II and IV depict values that would be classified differently by the two different statistical approaches. Hence, stimulation band noises with center frequencies exist at which 1 out of 4 animals was classified wrong ($\Delta PPI_{mean} < 0 \cap \Delta PPI_{fullc.} > 0 \cup \Delta PPI_{mean} > 0 \cap \Delta PPI_{fullc.} > 0$). This full combinatorial approach could be used to define a p-value according to Poe and colleagues, describing the p-value as fraction of the number of ΔPPI values >0 and all ΔPPI values (Poe et al. 2005).

4 Further examples for ratio distributions

In addition to the data exemplarily shown for one animal in Fig. 2 of the main text, the Suppl. Fig. 5 gives examples for ASR ratio distributions of another four animals. The Gaussian distribution in no case was a good approximation of the data. In all cases a lognormal distribution was the best choice to describe the data.

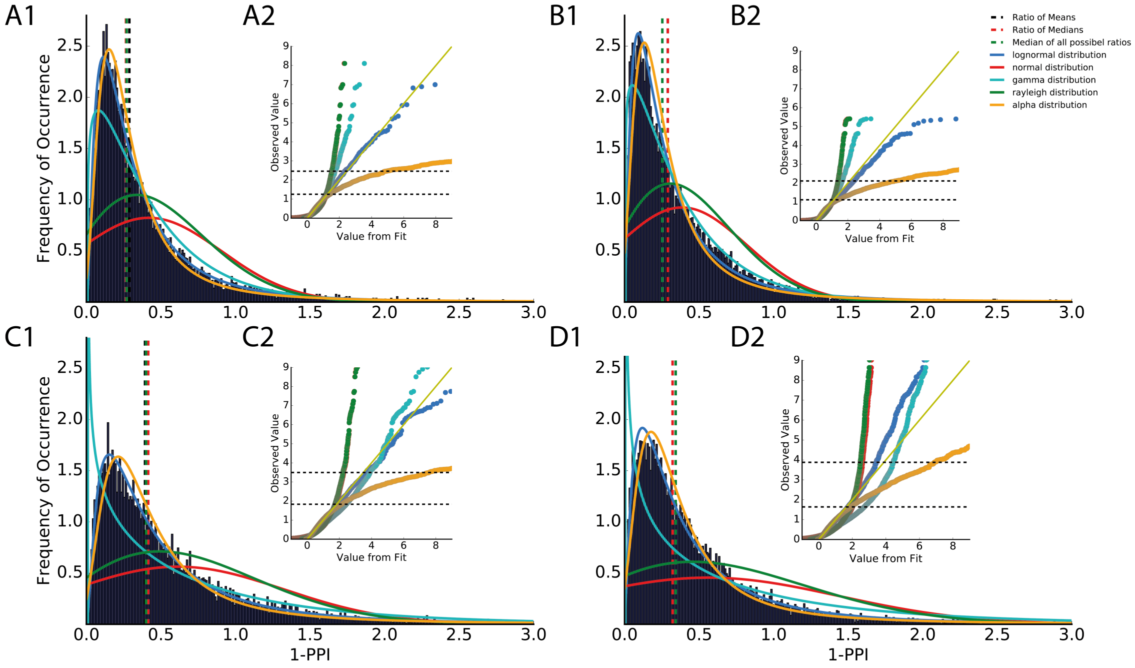

Supplementary Figure 5: **Lognormal distributed startle ratios**
Distribution of the ASR amplitude ratios from four animals (A to D) arising from all combinations of ASR amplitudes measured during gap and no-gap conditions (A1, B1, C1; D1). The Gaussian as well as the Rayleigh distribution in none of the cases was a valid approximation of the data. Alpha, gamma and lognormal distributions fit better. The q-q-plots (A2, B2, C2, D2, cf. Fig. 2 of the main text) provide evidence that in most cases a lognormal distribution fits best. However, for quantiles higher than 95% the data are not well-described by the lognormal distribution anymore.

5 Simulation study

The following section explains the application of the inferential statistics to simulated data sets. As already described in the Results section of the main text, the logarithmized startle amplitudes $\log(A)$ are nearly Gaussian distributed. Suppl. Fig. 6A shows an example of the underlying distributions of logarithmized gap amplitudes $\log(A_{\text{gap}})$ (cyan, $\mu_{gap} = 0$ and $\sigma_{\text{gap}} = 2$) and logarithmized no-gap amplitudes

$\log(A_{\text{nogap}})$ (red, $\mu_{\text{nogap}} = 1$ and $\sigma_{\text{nogap}} = 4$). From this underlying distributions samples of size n_{gap}=5 and n_{nogap}=10 were drawn. Suppl. Fig. 6B shows the mean values (10^6 repetitions) calculated from the drawn samples. Obviously the mean values are Gaussian distributed with the same mean μ as the underlying distribution and a standard deviation of $\sigma/\sqrt{n}$, i.e. the standard error of means (Suppl. Fig. 1B). From every repetition, the values of the two drawn samples with size n_{gap} and n_{nogap} were combined in every permutation and their difference

$$\begin{aligned} \Delta L &= \log(1 - PPI) \\ &= \log(A_{\text{gap}}) - \log(A_{\text{nogap}}) \end{aligned} \tag{1}$$

was calculated for all possible permutations (full combinatorial). From these permutations, the mean value was obtained, which corresponds to the mean pre pulse inhibition ratio.

It can be shown that the mean of the difference of all permutations equals the difference of the means:

$$\begin{aligned} &\text{mean}((\text{Fullc.}(L_{gap}, L_{nogap})) \\ &= \frac{1}{n_{\text{nogap}} \cdot n_{\text{gap}}} \cdot \left[\sum_{i=1}^{n_{\text{gap}}} \sum_{j=1}^{n_{\text{nogap}}} \left(L_i^{\text{gap}} - L_j^{\text{nogap}} \right) \right] \\ &= \frac{1}{n_{\text{nogap}} \cdot n_{\text{gap}}} \cdot \left[\sum_{i=1}^{n_{\text{gap}}} \left(n_{\text{nogap}} \cdot L_i^{\text{gap}} - \sum_{j=1}^{n_{\text{nogap}}} L_j^{\text{nogap}} \right) \right] \\ &= \frac{1}{n_{\text{nogap}} \cdot n_{\text{gap}}} \left[\sum_{i=1}^{n_{\text{gap}}} (n_{\text{nogap}} \cdot L_i^{\text{gap}} - n_{\text{nogap}} \cdot \text{mean}(L_{\text{nogap}})) \right] \\ &= \frac{1}{n_{\text{nogap}} \cdot n_{\text{gap}}} \cdot \left[n_{\text{nogap}} \sum_{i=1}^{n_{\text{gap}}} L_i^{\text{gap}} - n_{\text{gap}} \cdot n_{\text{nogap}} \cdot \text{mean}(L_{\text{nogap}}) \right] \\ &= \frac{1}{n_{\text{nogap}} \cdot n_{\text{gap}}} \cdot [n_{\text{nogap}} \cdot n_{\text{gap}} \cdot \text{mean}(L_{\text{gap}}) - n_{\text{gap}} \cdot n_{\text{nogap}} \cdot \text{mean}(L_{\text{nogap}})] \\ &\text{mean}(L_{\text{gap}}) - \text{mean}(L_{\text{nogap}}) \end{aligned} \tag{2}$$

Suppl. Fig. 6C shows the distribution of these means, obtained for the 10^6 repetitions. The obtained distributions of the simulated data were compared with the theoretically derived distributions. The good agreement of those two confirms the theoretical derivations.

In a next step, the same procedure was used to simulate the post acoustic trauma measurements. The according sample sizes $n_{\text{gap pre}} = 5$, $n_{\text{nogap pre}} = 10$, $n_{\text{gap post}} = 10$, $n_{\text{nogap post}} = 5$ were used. The according standard deviation of the logarithmized amplitudes are set to: $\sigma_{gap\ pre} = 2$, $\sigma_{nogap\ pre} = 4$ (according to Suppl. Fig. 6) and $\sigma_{gap\ pre} = 2$, $\sigma_{nogap\ pre} = 5$.

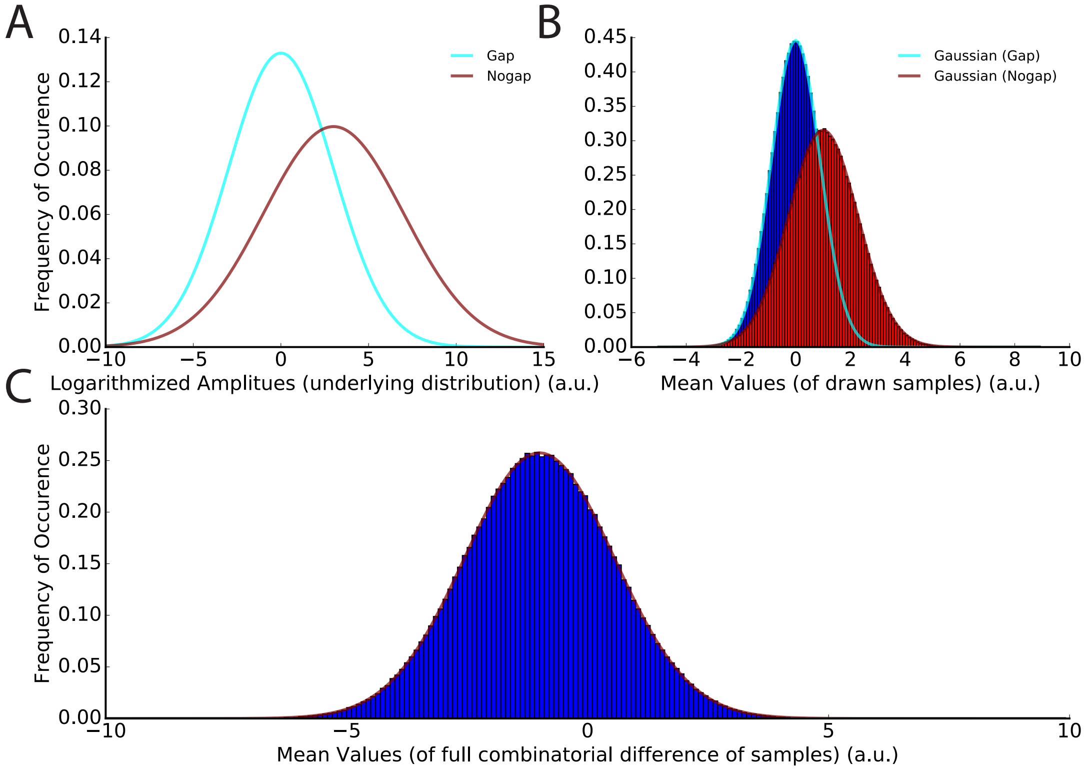

Supplementary Figure 6: **Evaluation of the extended statistics based on the Welch-Test (part I)**

A: Source distributions for the simulation, namely the logarithmized gap amplitudes (cyan, μ=0, σ=2) and no-gap amplitudes (red, μ=1, σ=4). B: Distribution of the mean values when 10^6 subsamples of size $n_{\text{gap}} = 5$ (blue bars) and $n_{\text{nogap}} = 10$ (red bars) are drawn from the underlying distributions. The standard deviation of theses Gaussian distributions is the standard error of means. Simulations were in good agreement with the theoretical distribution (cyan and red lines). C: Distribution of the mean value of the differences between all gap to no-gap amplitudes drawn in each repetition (blue), which was also Gaussian distributed (red curve).

This choice should represent the general case of different sample sizes and accordingly different standard errors of means (Suppl. Fig. 7A) and no variance homogeneity. The difference of the log ratios was calculated as follows:

$$\begin{aligned}\Delta L_{\text{pre\_post}} &= L_{\text{pre}} - L_{\text{post}} \\ &= \text{mean}(\log(1 - PPI_{\text{pre}})) - \text{mean}(\log(1 - PPI_{\text{post}}))\end{aligned} \quad (3)$$

The difference of the mean ratios pre and post trauma was again Gaussian distributed as shown in Suppl. Fig. 7B. As shown in equ. 2 the difference of means equals the mean of the all combinatorial differences (full combinatorial).

The cyan line marks the Gaussian distribution with a standard deviation being equal to the standard error of the mean difference. However, when every $\Delta L_{\text{pre\_post}}$ was normed by the according standard error of means the resulting distribution was not Gaussian any more, but followed a Student's t-distribution (Suppl. Fig. 7C). For higher sample sizes the t-distribution becomes Gaussian again. The t-distribution has a slightly different shape than a Gaussian distribution with higher tails, which is important for the p-value calculation. For the cyan t-distribution the Welch-approximation (Welch, 1938; 1947) was used and the sample sizes were set to the effective sample size. The red curve represents a more conservative estimation using the minimum sample size ($n = 5$) to calculate df (degrees of freedom). The green curve, in contrast, represents a less conservative estimation using the maximum sample size ($n = 10$) to calculate the df. The integral function of the t-distribution is shown in Suppl. Fig. 7E. This function is used to determine the p-value. Suppl. Fig. 7F shows the fraction of significant classified samples (animals with significant PPI change) for the different types of df-estimations. That means when the samples are drawn from the same distribution, so that there is no real difference between the mean value of the pre and the post ratios the Welch-test for the compound variable (log ratios) leads to 3.9 % significant samples (conservative, used sample size set to minimum sample size), the proposed method (using effective sample size) leads to 4.7 % significant values and the most liberal estimation (maximum sample size) leads to 5.9 % significant values. In other words, the method described in the paper leads to even a slight overestimation of the p-value: namely 4.7 % of the cases the zero hypothesis is wrongly rejected for a pre-defined significance level of 5 %. Thus, the method is a good but slightly conservative approach to estimate the significance level of GPIAS data.

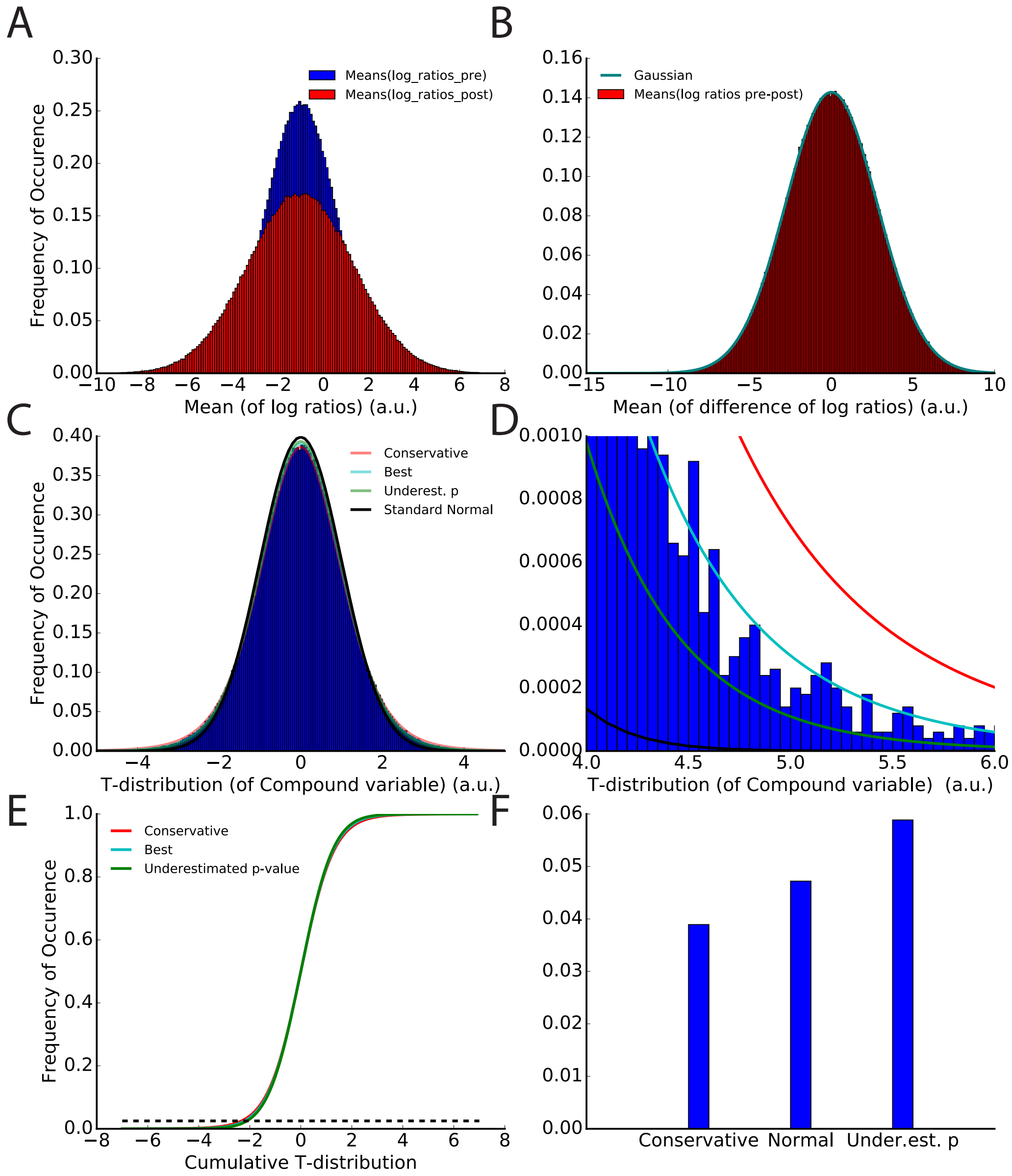

Supplementary Figure 7: **Evaluation of the extended statistics based on the Welch-Test (part II)**

A: Distribution of the means of the log ratios of pre and post trauma condition (cf. Suppl. Fig. 6, histogram in Suppl. Fig. 6 equals blue histogram in A); B. Difference of the means of the pre and post ratios. C: Normed difference of mean ratios (normed by the respective combined standard error of the mean difference); These values are not Gaussian but t-distributed. The curves represent the respective t-distribution. For the calculation of the degrees of freedom the Welch approximation was applied. D: Zoom to the edges (of C) of the t-distributions and the histogram. E: Integral function of the t-distributions: (black dashed line: 2.5 %, used for two-sided test) F: Fraction of significant classified samples. The value should equal 5 %. The plot shows that even for the use of the effective sample size the p-values are slightly overestimated and consequently the fraction of significantly false classified data slightly underestimated. Note that a two-sided test was applied (includes significant negative and positive changes of gap detection ability).

6 Classification of T (tinnitus) animals: significance criterion compared to effect size threshold

The following simulation compares the difference of the classification of T-animals based on a significance criterion versus setting of an effect size threshold. As shown in Fig. 5 of the main text the effect sizes (corrected Hedges g) as well as the ΔPPI are continuously distributed across animals. Hence, in a certain range all different values for the effect size could possibly occur. The following simulation was based on this observation. As described in the main text the fraction of T-classified animals for this continuous distribution of effect sizes (or ΔPPIs) across animals monotonically increases with increasing number of measurement repetitions. In other words, the increase of the number of measurement repetitions does not lead to a more exact estimation of the mean value but leads to systematic increase of the number of T-classified animals. Nevertheless, as the effect sizes are continuously distributed, they possibly could correlate with the perceived tinnitus intensity. Thus, a threshold should be set, which represents the effect size correlated with a particular tinnitus intensity, leading to a remarkable change of the PPI post acoustic trauma.

The following simulation is based on 33 simulated data sets (refer to 33 hypothetical animals). The simulated effect sizes were in the range [-1.6, 1.6] with a step width of 0.1 (each animal one of these effect sizes). Hence, the effect sizes were uniformly distributed across the complete interval. The effect sizes were used to calculate the mean difference between post and pre trauma condition of the underlying distributions of (underlying distributions of logarithmized gap and no-gap amplitudes) these hypothetical animals. That means, the animals are represented by an effect size and a difference of log pre and post trauma ratio.

This calculated mean difference ($meandiff$) was used to shift the post trauma log gap amplitude distribution. That means if infinitely large samples of the four log amplitude distributions ($\mathrm{N}(\mu_{gap\ pre}, \sigma_{gap\ pre})$, $\mathrm{N}(\mu_{nogap\ pre}, \sigma_{nogap\ pre})$, $\mathrm{N}(\mu_{gap\ post}, \sigma_{gap\ post})$, $\mathrm{N}(\mu_{nogap\ post}, \sigma_{nogap\ post})$) are drawn, the effect size will approach the value of the respective mean difference. The underlying distributions of the log amplitudes equal the distributions used for the previous simulations. That means for the log ratios the underlying distributions are of the form: $\mathrm{N}(\mu_{pre} = -1, \sigma_{pre} = \sqrt{20})$ and $\mathrm{N}(\mu_{post} = -1 + meandiff, \sigma_{post} = \sqrt{29})$.

To simplify the simulation the sample sizes (measurement repetitions) were set to equal values ($n = n_{\mathrm{pre\_gap}} = n_{\mathrm{pre\_nogap}} = n_{\mathrm{post\_gap}} = n_{\mathrm{post\_nogap}} \in \{3, 4, 5, 6, 7, 8, 9, 10, 11, 12, 13, 14, 15, 20, 50, 100, 200\}$). This is necessary to enable for a systematic analysis of the fraction of T animals as a function of applied measurement repetitions (number of repetitions of each stimulus). The four drawn samples of size n were evaluated using the same function (Welch-test for compound variable and evaluation of Hedges g) as used for the real data. The procedure was repeated 10^4 times for each sample size (n) to estimate the variance of the results.

Suppl. Fig. 8 shows that the fraction of T-classified animals based on a signifi-

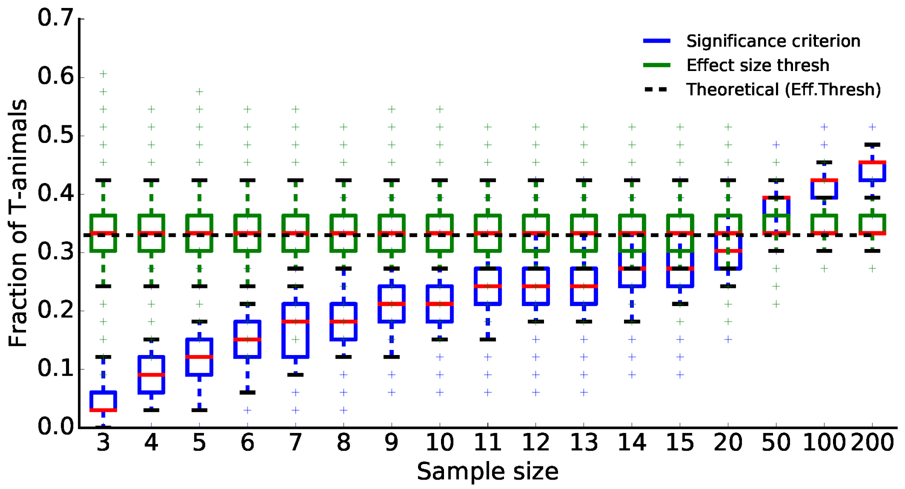

Supplementary Figure 8: **Classification of T animals**
The fraction of T animals as a function of applied measurement repetitions (n); (red lines: medians, bars: quartiles, whisker: 5 %-95 % quantiles, markers: outliers). The significance criterion (blue) leads to a monotonically increase of the fraction of T animals. (Note that the p-criterion means $p < 0.05$ and effect size < 0) The effect size criterion (green) leads to a saturation at a constant values (theoretical value). Note that for the complete analysis only one stimulus frequency was assumed.

cance criterion (blue) increases monotonically with increasing number of measurement repetitions. In contrast setting of a constant effect size threshold leads to a saturation of the fraction of classified T-animals (green). The black dashed line marks the fraction of T-animals for the ideal case of of an infinite amount of measurement repetitions (n). That means the number of effect sizes smaller than -0.5 for the ideal case. The fact that the green boxes saturate at this values proves the correctness of the simulation. Furthermore, it can be shown that with rising number of measurement repetitions (n) the variance of the fraction of T animals based on the effect size criterion decreases.

In conclusion, although the paper describes a valid method for estimating the p-value, this measure is not a valid choice to determine the number of T animals. In other words, the p-criterion ($p < 0.05$ and a reduction of the gap detection ability) leads to a shift of the effect size necessary to classify an animal as T animal for a given number of measurement repetitions.

7 References

Poe, G.L., Giraud, K.L., and Loomis, J.B. (2005). Computational methods for measuring the difference of empirical distributions. American Journal of Agricultural Economics 87, 353-365.

Welch, B.L. (1938). The significance of the difference between two means when the population variances are unequal. Biometrika 29, 350-362.
Welch, B.L. (1947). The generalization of student's' problem when several different population variances are involved. Biometrika 34, 28-35.
